# DUF6383-domain containing protein from *Parabacteroides distasonis* predicts survival in patients with alcohol-associated hepatitis

**DOI:** 10.1080/29933935.2026.2728306

**Published:** 2026-10-28

**Authors:** Henriette Kreimeyer, Carlos Gonzalez, Marcos F. Fondevila, AlcHepNet Investigators, Ali Canbay, Peter Stärkel, David J. Gonzalez, Bernd Schnabl

**Affiliations:** a Department of Medicine, University of California San Diego, La Jolla, CA, USA; b Department of Medicine, Knappschaft Kliniken Universitätsklinikum Bochum, Ruhr University Bochum, Bochum, Germany; c Department of Pharmacology, University of California San Diego, La Jolla, CA, USA; d Skaggs School of Pharmacy and Pharmaceutical Sciences, University of California San Diego, La Jolla, CA, USA; e Roster for authorship listed in Supplementary Material; f Department of Hepatology and Gastroenterology, Cliniques Universitaires Saint Luc, Brussels, Belgium; g Department of Medicine, VA San Diego Healthcare System, San Diego, CA, USA

**Keywords:** Metaproteomics, microbiota, microbiome, alcohol-associated liver disease, alcoholic liver disease

## Abstract

Intestinal dysbiosis is a hallmark of alcohol-associated liver disease (ALD) characterized by a higher abundance of unfavorable species and a reduction of beneficial species. In this study, we aimed to analyze the bacterial proteome of stool samples from patients with alcohol-associated hepatitis (AH) (*n* = 80), alcohol use disorder (AUD; *n* = 20), and controls (*n* = 19) from an international multicenter cohort using quantitative tandem mass tag (TMT)-based metaproteomic profiling. We validated our results in an independent multicenter cohort of 72 patients with AH at the genomic level using qPCR. We detected 2,364 bacterial proteins and identified distinct metaproteomic signatures separating controls, patients with AUD, and AH. Patients with AUD and AH were characterized by depletion of Firmicutes-derived proteins and enrichment of proteins from Bacteroidetes, Proteobacteria, and Actinobacteria. Eighteen bacterial proteins increased with disease severity, many with multifunctional “moonlighting” roles, including enolase and hexokinase. DUF6383 domain-containing protein from *Parabacteroides distasonis* is correlated with histological injury, inflammatory cell infiltration, and is linked to neutrophil degranulation in the host. Patients with a high abundance of DUF6383 domain-containing protein had significantly lower 90-day survival, and this association was validated by qPCR at the genomic level in a second cohort. Our findings show that microbial proteins are associated with disease severity and progression, and that DUF6383 domain-containing protein from *P. distasonis* is a potential prognostic biomarker for AH.

## Introduction

Alcohol-associated liver disease (ALD) is a major cause of liver-related morbidity and mortality worldwide, ranging from benign hepatic steatosis to alcohol-associated hepatitis (AH), which 10–20% of patients with alcohol use disorder (AUD) develop.^1-4^ AH is marked by an acute onset of jaundice after heavy and chronic alcohol use.^5^ Patients frequently develop infections, systemic inflammatory response syndrome, and renal failure, which influences 90-day mortality.^6^ Short-term survival is low with rates of 69–81.5% at day 28 and 62.5–69.1% at day 90.^7-10^

Clinical tools and scoring systems aid in identifying patients who may benefit from more intensive therapy, with the Model for End-Stage Liver Disease (MELD) score demonstrating the highest predictive accuracy.^10^ ^,^ ^11^ Beyond alcohol abstinence, therapeutic options include corticosteroids, which improve short-term but not long-term mortality, and early liver transplantation as the only definitive intervention^8^ ^,^ ^12-14^

Alcohol induces intestinal inflammation via activation of macrophages and monocytes in the jejunum and release of tumor-necrosis factor.^15^ This leads to intestinal barrier dysfunction, allowing the translocation of microbial-associated molecular patterns (MAMPs) into the systemic circulation, thereby enhancing the systemic immune response and liver inflammation.^3^ ^,^ ^16^ Alcohol also leads to gut dysbiosis, including shifts in bacterial, viral, and fungal populations, characterized by decreased bacterial and fungal diversity and increased viral diversity.^17-19^ Pathogenic bacteria like *Enterococcus, Prevotella*, and *Veillonella* increase while beneficial bacteria like *Akkermansia muciniphila, Bifidobacterium,* and *Faecalibacterium prausnitzii* decrease^18-20^

Most studies have focused on taxonomic profiling, which reveals microbial presence but not their functional activity. Metaproteomics enables large-scale characterization of microbial protein expression in complex samples, providing a snapshot of active microbial functions. Unlike DNA- or RNA-based methods, metaproteomics directly identifies proteins that execute metabolic and signaling roles, reflecting the functional state of the microbiome. This approach can reveal microbial factors influencing disease progression and identify novel biomarkers and therapeutic targets. We recently characterized the fecal host proteome in patients with AH and observed that proteins associated with neutrophil degranulation were linked to disease severity and survival.^21^ Myeloperoxidase could predict 60-day survival.^21^

Here, we analyzed quantitative TMT-based metaproteomics of fecal samples from controls, patients with AUD, and AH, focusing on the bacterial metaproteome. Our aim was to identify a microbial protein signature of patients with ALD and to identify a biomarker.

## Materials and methods

### Patient cohorts

Fecal samples were derived from two different cohorts, which have been described previously.^21^ Both studies were conducted with the approval of the ethics committee of each participating institution, and written informed consent was obtained from every participant prior to enrollment. Stool samples were collected at the time of hospital admission.

#### Metaproteomics cohort:

Details of this cohort have been reported.^18^ ^,^ ^20-23^ Participants with AH were enrolled between June 2014 and May 2017 as part of a prospective, international, multicenter study conducted across eleven hospitals spanning five global regions (Integrated Approaches for Identifying Molecular Targets in Alcoholic Hepatitis (InTeam) Consortium, ClinicalTrials.gov identifier: NCT02075918). We selected 80 patients with AH to include in our analysis, accompanied by 20 patients with AUD and 19 controls. Baseline demographic and clinical characteristics of the AH cohort are summarized in Table 1.

**Table 1.** Clinical characteristics of patients with AH (InTeam) and AUD in the metaproteomics analysis.

Variable	Patients with alcohol-associated hepatitis *n* = 80	Patients with alcohol use disorder *n* = 20	*P*-value
**Clinical parameters**			
Age (years)	49.95 (30–68), *n* = 77	46.27 (35–66), *n* = 15	0.22
Sex (male), *n* (%)	52 (67.53%), *n* = 77	5 (45.45%), *n* = 11	0.18
Weight (kg)	83.15 (49–153), *n* = 67	77.88 (45.2–120), *n* = 15	0.5
Height (m)	1.71 (1.55–1.88), *n* = 69	1.61 (1.55–1.82), *n* = 15	0.9
BMI (kg/m2)	28.32 (19–48.29), *n* = 65	26.74 (17.4–39.18), *n* = 15	0.4
**Laboratory parameters**			
Albumin (g/dl)	2.45 (1.3–4.3), *n* = 74	4.47 (3.8–5.0), *n* = 15	<0.001
ALP (IU/L)	196.49 (21.2–456), *n* = 76	107.87 (39–199), *n* = 15	<0.001
ALT (IU/L)	52.45 (15–174), *n* = 77	91.33 (13–236), *n* = 15	0.084
AST (IU/L)	150.05 (41–406), *n* = 77	126.27 (14–442), *n* = 15	0.089
Bilirubin (mg/dl)	16.48 (3.06–36.62), *n* = 77	0.62 (0.3–1.4), *n* = 15	<0.001
Creatinine (mg/dl)	1.08 (0.31–8.14), *n* = 77	0.75 (0.44–0.94), *n* = 15	0.5
GGT (IU/L)	477.41 (33–3145), *n* = 37	345.4 (17–931), *n* = 15	0.9
INR	2 (1–4.4), *n* = 76	1 (0.88–1.23), *n* = 13	<0.001
Cholesterol (mg/dl)	134.86 (0.54–468), *n* = 19	217.5 (151–331), *n* = 8	0.004
Triglycerides (mg/dl)	121.77 (0.75–611), *n* = 19	219.12 (52–480), *n* = 8	0.053
**Non-invasive fibrosis assessment**			
Fibroscan *n* = 15			
F0-F1		10 (67%)	
F2		3 (20%)	
F4		2 (13%)	
**Clinical presentation and treatment at admission**			
Steroids, *n* (%), *n* = 77	13 (16.88%)		
Antibiotics, *n* (%), *n* = 77	3 (3.9%)		
Infections, *n* (%), *n* = 58	12 (21%)		
Alcohol consumption at day 90, *n* = 27			
Abstinent, *n* (%)	24 (88.89%)		
Reduced alcohol consumption, *n* (%)	2 (7.4%)		
Continued alcohol abuse, *n* (%)	1 (3.7%)		
Stage of fibrosis, *n* (%), 0/1/2/3/4, *n* = 40	1 (2.5)/0 (0)/5 (13)/6 (15)/28 (70)		
**Clinical scores and outcome**			
MELD, mean (range)	24.97 (11.7–40), *n* = 76		
MELDNa, mean (range)	27.55 (15.29–40), *n* = 76		
Child‐Pugh stage A/B/C, *n* (%), *n* = 75	1 (1.3%)/18 (24%)/56 (75%)		
30-day-mortality, *n* (%), *n* = 71	9 (12.68%)		
90-day-mortality, *n* (%), *n* = 72	16 (22.22%)		

Values are presented as mean and range in parentheses. The number of patients for whom data was available is indicated for each group column. *P*-values were calculated with Wilcoxon-Test for continuous variables and Exact Fisher’s Test for categorical variables.

BMI, body mass index; ALP, Alkaline phosphatase; ALT, alanine aminotransferase; AST, aspartate aminotransferase; GGT, gamma-glutamyl-transferase; INR, international normalized ratio; MELD, model of end-stage liver disease.

Diagnosis of AH was based on self-reported active alcohol abuse in the last three months (>50 g/day for men and >40 g/day for women) and clinical presentation. If clinically indicated, a liver biopsy was performed. Further eligibility criteria were biochemical features of liver injury with aspartate amino transferase (AST) exceeding alanine aminotransferase (ALT) and total serum bilirubin levels greater than 3 mg/dl in the last three months. Patients were excluded if they had evidence of autoimmune liver disease (ANA > 1/320), chronic viral hepatitis, hepatocellular carcinoma, complete portal vein thrombosis, extrahepatic terminal disease, or pregnancy.

The AUD cohort was recruited independently in Brussels, Belgium, from individuals admitted for elective inpatient alcohol rehabilitation. All met the Diagnostic and Statistical Manual of Mental Disorders, Fifth Edition criteria for AUD and reported sustained alcohol consumption exceeding 60 g/day for more than one year, with continued drinking until admission.^24^ While liver disease was present in some participants, none met the criteria for AH. Patients with use of antibiotics or immunosuppressive medication for two months before enrollment, diabetes, inflammatory bowel diseases, non-alcohol-related liver disease, or clinically significant cardiovascular, pulmonary, or renal comorbidities were excluded.

Control participants have been described previously.^25^

#### Validation cohort:

Details of this cohort have been described.^21^ We analyzed data and biological samples from 72 individuals enrolled through the Alcoholic Hepatitis Network (AlcHepNet, NCT04072822). Enrollment occurred between May 2019 and May 2022 at eight clinical centers in the United States, including Cleveland Clinic, Indiana University, Mayo Clinic, University of Louisville, Beth Israel Deaconess Medical Center Boston & University of Massachusetts, University of Pittsburgh, University of Texas Southwestern, and Virginia Commonwealth University.

Eligible participants were at least 21 years old, had a clinical diagnosis of AH, and had a serum total bilirubin of >3 mg/dl. Additionally, they or a guardian had to be able to understand and provide written informed consent. Subjects were excluded if they were unable to understand or comply with study procedures based on the judgment of the investigator. Although the study protocol allowed the re-enrollment of patients presenting with a new episode of AH occurring at least 24 weeks after a previous episode (up to four total enrollments), no repeat re-enrollments were included in the present analysis. Subjects were excluded if they had alternative causes of liver disease, including hemochromatosis, autoimmune hepatitis, Wilson disease, nonalcoholic fatty liver disease, or acute viral hepatitis, or if they were pregnant or breastfeeding. Chronic infection with hepatitis B virus, hepatitis C virus, or HIV did not exclude participation in the absence of clinically significant liver disease. Within this cohort, one participant had resolved hepatitis C infection (antibody positive, RNA negative), and three had evidence of prior hepatitis B exposure based on surface antibody positivity without detectable surface antigen.

### Protein extraction, tandem mass tag (TMT) proteomics, mass spectrometry data acquisition and metaproteomics analysis

Samples for metaproteomics and downstream analysis were processed as described.^21^ TMT-based proteomics enables relative quantification of protein abundances. Briefly, acquired spectra were searched against a FASTA sequence database.^26^ Only high-confidence protein matches (protein-level quality scoring filtered q < 0.01) were included in downstream analyses. Additionally, proteins identified were subsequently blasted against two different databases using blast2go and eggnogger for functional annotation. For this study, we focused on bacterial proteins. Results are calculated based on these accessions. Data was log-transformed and normalized using median normalization. All .RAW data and .MZML data were deposited at massive.ucsd.edu, Study ID: MassIVE MSV000094610.

### Fecal DNA extraction and qPCR

The genomic quantity of DUF6383 domain-containing protein was determined in fecal DNA using qPCR. DNA was extracted from human fecal samples with the QIAmp Fast DNA Stool Mini Kit (QIAGEN, Hilden, Germany; Cat. No.: 51604).^27^ We used the DUF6383 domain-containing protein sequence provided by the NIH gene database and matched it against the sequence of the protein. The primers used were designed using Primer3 and the Primer blast tool of the NIH (Primer Forward: GAGCCAAGCCCTTGATCCAT, Reverse: GCTAGCCGCTATGAGTTGGT). qPCR was performed with iTaq Universal SYBR Green Supermix (Biorad, Hercules, CA, USA, Cat No.: 7125125) using the QuantStudio™ 5 Real-Time PCR System (Thermo Fisher Scientific, WA, USA).

### Statistical analysis

Results are reported as median and range unless otherwise specified. Comparisons between two groups were conducted using Mann-Whitney-Wilcoxon rank sum test for continuous variables or Fisher’s exact test for categorical variables, as appropriate. Comparisons across three or more groups were conducted using the Kruskal–Wallis Test for continuous variables or Fisher’s exact test for categorical variables, with correction for multiple testing with false discovery rate or Benjamin–Hochberg. For testing groups adjusted to other covariates, ANCOVA was performed. All the statistical tests were two-sided.

Normalization, logarithmic transformation, and differential abundance of proteins were carried out using the “protti” package in R. PCA was performed using the “base” and “forcats” packages in R. Group differences in the PCA space were assessed by calculating the dissimilarity index using the method of Bray–Curtis, followed by a Permutational Multivariate Analysis of Variance Using Distance Matrices.^28^

Heatmaps were generated by hierarchical clustering using the “corrplot” package in R and the “ward.D2” method. Pearson correlation was calculated with the “Hmisc” package in R. Correlations > 0.2 and < −0.2 were regarded as clinically relevant when significant. Survival analysis was conducted with Kaplan-Meier curves and the log-rank test. Patients were censored at the time point when last seen alive. The time point of liver transplantation was regarded as the time point of death.

Patients were classified into two groups depending on the abundance of a protein using maximal selected rank statistics. Functional enrichment analysis was performed with the “ClusterProfiler” or “protti” package in R.^29^ ^,^ ^30^ Statistical analysis was performed using R statistical software, R version 4.3.1 (2023).

## Results

### Bacterial metaproteomic profiles show distinct differences in fecal samples from controls, patients with AUD and AH

Fecal samples of 19 controls, 20 patients with AUD, and 80 patients with AH were analyzed for bacterial proteins using TMT-based metaproteomics. Patients with AH were enrolled through the InTeam international multicenter consortium. The cohort has been characterized and published in our previous paper characterizing the host proteome.^21^ The mean Model for End-Stage Liver Disease (MELD) score among patients with AH was 24.97, and the observed 30-day and 90-day mortality rates were 12.68% and 22.22%, respectively (Table 1).

Acquired spectra were mapped to proteins from a metagenomically generated protein database, as described.^26^ After quality filtering, 2,364 proteins were present in the AUD and AH groups, but only 1,603 were present in the control group. The metaproteomic signature of controls, patients with AUD and AH are significantly different between all three groups following principal component analysis (PCA) using a permutational multivariate analysis of variance (PERMANOVA) (Figure 1A). AUD and AH groups were also significantly different from controls as well as from each other (Control vs. AH, *p* = 0.003, Control vs. AUD, *p* = 0.003, AUD vs. AH, *p* = 0.03) (Figure 1A). There is no significant difference in the fecal protein signature between patients who received steroids compared with no steroids at the time of stool collection, antibiotics vs. no antibiotics at the time of stool collection, or who had an infection (Supplemental Figure 1A–C).

**Figure 1.**
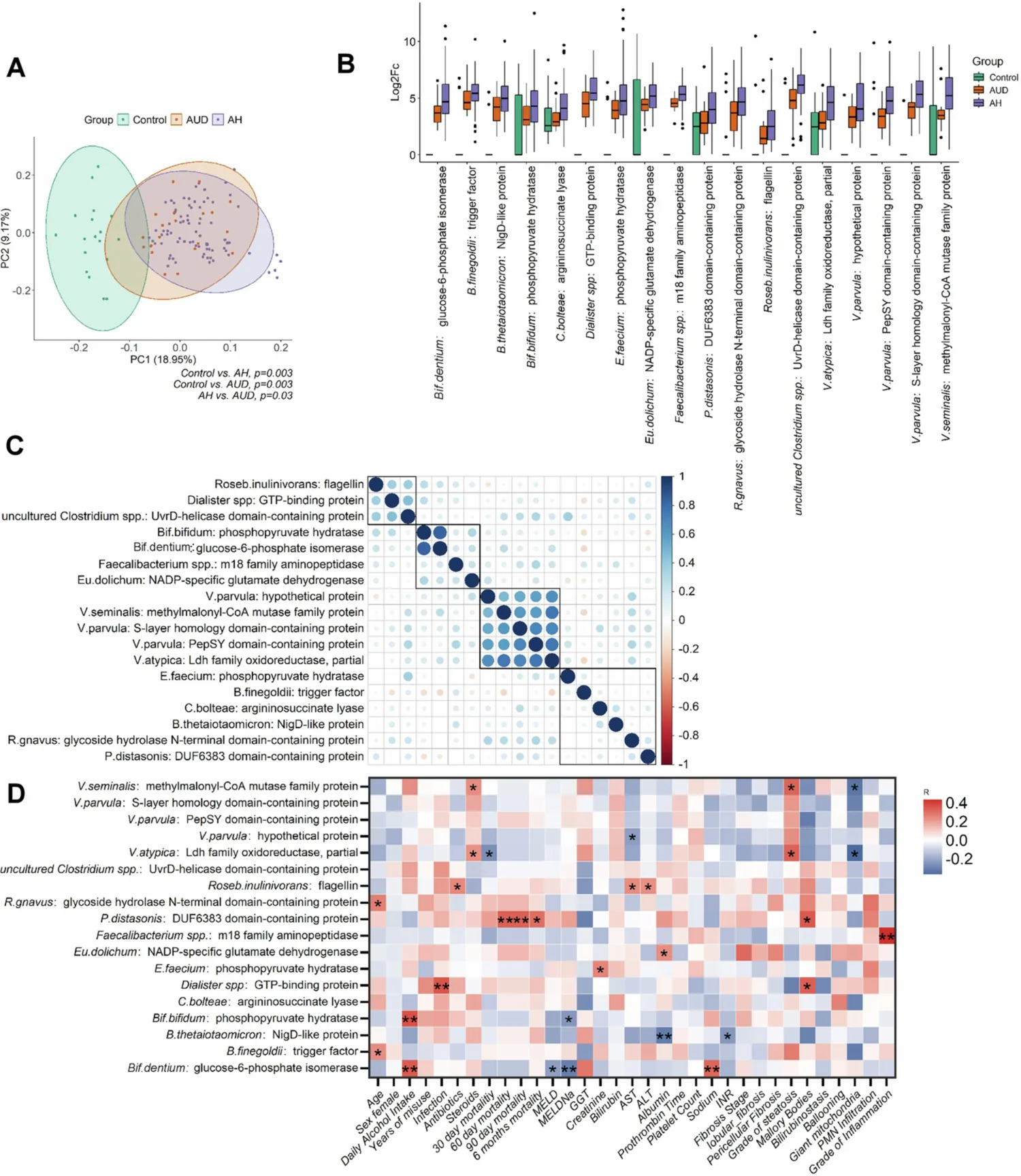
Bacterial protein patterns are different in the feces between controls, patients with alcohol use disorder (AUD), and patients with alcohol-associated hepatitis (AH). Characterization of bacterial proteins was performed from fecal samples of controls (Control, *n* = 19), patients with alcohol use disorder (AUD; *n* = 20), and patients withalcohol-associated hepatitis (AH, InTeam; *n* = 80). (A) Bacterial proteins in fecal samples in controls, AUD, and AH groups (*P* = 0.001 PERMANOVA test [^28^]; AH vs. AUD *P*_*adj.*_ = 0.03, AH vs. healthy *P*_*adj.*_ = 0.003, AUD vs. control *P*_*adj.*_ = 0.003. *P*-values were calculated using the dissimilarity index calculated by the method of Bray-Curtis and Permutational Multivariate Analysis of Variance Using Distance Matrices. (B) Protein abundance of the 18 detected proteins, which showed an increase with disease severity and a significant difference between all three groups as calculated by Kruskal–Wallis test, and a significant difference between AH and AUD patients following Wilcoxon rank sum test. *P-*values for Kruskal–Wallis Test and Wilcoxon rank sum test are listed in Supplemental Table 1. *Bif. dentium*, *Bifidobacterium dentium*; *B. finegoldii, Bacteroides finegoldii; B. thetaiotaomicron, Bacteroides thetaiotaomicron; Bif. bifidum, Bifidobacterium bifidum; C. bolteae, Clostridium bolteae; E. faecium, Enterococcus faecium; Eu. dolichum, Eubacterium dolichum; P. distasonis, Parabacteroides distasonis; R. gnavus, Ruminoccous gnavus; Roseb. inulinivorans, Roseburia inulinivorans; V. atypica, Veillonella atypica; V. parvula, Veillonella parvula; V. seminalis, Veillonella seminalis.* (C) A heatmap of bacterial proteins in fecal samples was clustered using hierarchical clustering. The x-axis is the same as the y-axis. *P-*values were calculated using Pearson correlation. (D) Pearson correlation of the 18 differentially expressed proteins from Figure 1B with histological, clinical, and laboratory parameters. *P < 0.05; **P < 0.01.

To better characterize the proteins that drive these differences, we filtered all proteins using three steps. First, we filtered for all proteins that show a significant difference between all three groups (1,261 proteins). Then we filtered proteins that also showed a significant difference between patients with AUD and AH (241 proteins). Finally, we filtered for a progressive increase or decrease in relation to the progression of alcohol-associated liver disease (169 proteins). From those, 18 proteins increased with disease severity, and 151 decreased with disease severity. Five of the increasing proteins were produced by *Veillonella*. Two proteins each are produced by *Bifidobacterium, Clostridium,* and *Bacteroides*. Phosphopyruvate hydratase, also called enolase, was increased in *Bifidobacterium* and *Enterococcus*. Other bacteria that produced proteins that increased were *Roseburia, Dialister, Eubacterium, Faecalibacterium, Ruminoccocus* and *Parabacteroides* (Figure 1B, Supplemental Table 1). Notably, proteins from *Veillonella seminalis, Veillonella atypica, Parabacteroides distasonis, Eubacterium dolichum, Clostridium. bolteae* and *Bifidobacterium bifidum* were present in controls, patients with AUD and AH, while all others were present only in patients with AUD and AH.

Several of the increased proteins are “moonlight” proteins, which exhibit more than one function. Enolase, for example, is part of the glycolysis pathway, but it is also described as a plasminogen activator in *Bifidobacterium spp*. On the surface of other bacteria, it binds to mucin, fibronectin, and laminin.^31^ Further functions include the fusion of vacuole membranes, response to stress conditions, components of the degradosome, and interaction with phospholipid metabolism.^32^ Another protein which is increased, glucose-6-phosphate isomerase (hexokinase), activates the inflammasome NLR family pyrin domain containing 3 (NLRP3).^32^

We applied Ward’s hierarchical clustering to assess whether there are connections among the proteins (Figure 1C). As expected, all proteins from *Veillonella* and *Bifidobacterium* were positively correlated with each other. Proteins from *Bifidobacterium* were positively correlated with the proteins from *Faecalibacterium spp.* and *Eu. dolichum*. Proteins from *Clostridium* and *Clostridium*-related bacteria, namely, *Ruminococcus, Dialister*, and *Clostridium,* formed another group that correlated positively with each other. Finally, the last group encompassed proteins that did not correlate with other proteins, namely, *Enterococcus faecium*, *B. finegoldii, C. bolteae, Bacteroides thetaiotaomicron*, *R. gnavus,* and *P. distasonis*.

In summary, our analysis revealed distinct fecal metaproteomic signatures across controls, patients with AUD, and AH, with specific bacterial proteins increasing with liver disease severity. These proteins cluster into functionally and taxonomically related groups, highlighting *Veillonella*, *Bifidobacterium*, and *Clostridium*-related taxa as key drivers of liver disease-associated changes.

### DUF6383 domain-containing protein can predict short-term survival in patients with AH

To understand the clinical relevance of these 18 proteins, we correlated them with clinical parameters and survival. DUF6383 domain-containing protein, whose function remains uncharacterized, produced by *P. distasonis,* was positively correlated with 60−, 90−, and 180-day mortality. While LDH family oxidoreductase was negatively correlated with 30-day mortality, hexokinase and enolase were negatively correlated with the MELD- and MELDNa score (Figure 1D). Notably, hexokinase and enolase produced by *Bifidobacterium* are positively correlated with daily alcohol consumption. GTP-binding protein from *Dialister spp.* is positively correlated with the occurrence of infections, and flagellin produced by *Roseburia* with the administration of antibiotics. All proteins from *Veillonella* were positively correlated with steatosis and negatively correlated with the level of giant mitochondria, but only the methylmalonyl-CoA mutase family protein produced by *V. seminalis* and Ldh family oxidoreductase produced by *V. atypica* showed significance. M18 family aminopeptidase by *Faecalibacterium spp.* is significantly correlated with the grade of inflammation. Patients with a high level of Mallory bodies show a significantly higher abundance of DUF6383 domain-containing protein from *P. distasonis* (*p* = 0.011) (Figures 1D and 2A) and GTP-binding protein from *Dialister spp.* (*p* = 0.024) (Figures 1D and 2B). Mallory bodies are misfolded proteins containing keratin 8 and 18, ubiquitinated proteins, chaperone and misfolded proteins, transglutaminase-2, and p62.^33^ ^,^ ^34^ They accumulate in diverse liver diseases but are especially enriched in AH.^34^ Their occurrence is related to impaired autophagy and indicates severe liver damage.^33^ We categorized patients into two groups depending on the abundance of DUF6383 domain-containing protein using max rank selected statistics. Patients with a high abundance of DUF6383 domain-containing protein had significantly lower survival on day 90 compared to patients with a low DUF6383 domain-containing protein abundance (Figure 2C). It is worth noting that there is no difference in the MELD- and MELDNa Score between patients with high and low DUF6383 domain-containing protein abundance (*p* = 0.74 and *p* = 0.37, respectively). Severe AH, as defined by a Maddrey Score ≥ 32, was present in 51 patients. DUF6383 domain-containing protein showed a trend of being higher in those patients but missed significance (AH: 3.5, SAH 3.93, *p* = 0.078, Figure 2D). There was no significant difference in DUF6383 domain containing protein levels in patients who received steroids or antibiotics at the time of sample collection after adjusting for the MELD Score (steroids: ANCOVA: F = 0.79, *p* = 0.69, antibiotics: ANCOVA: F = 1.14, *p* = 0.36) (Supplemental Figure 2).

**Figure 2.**
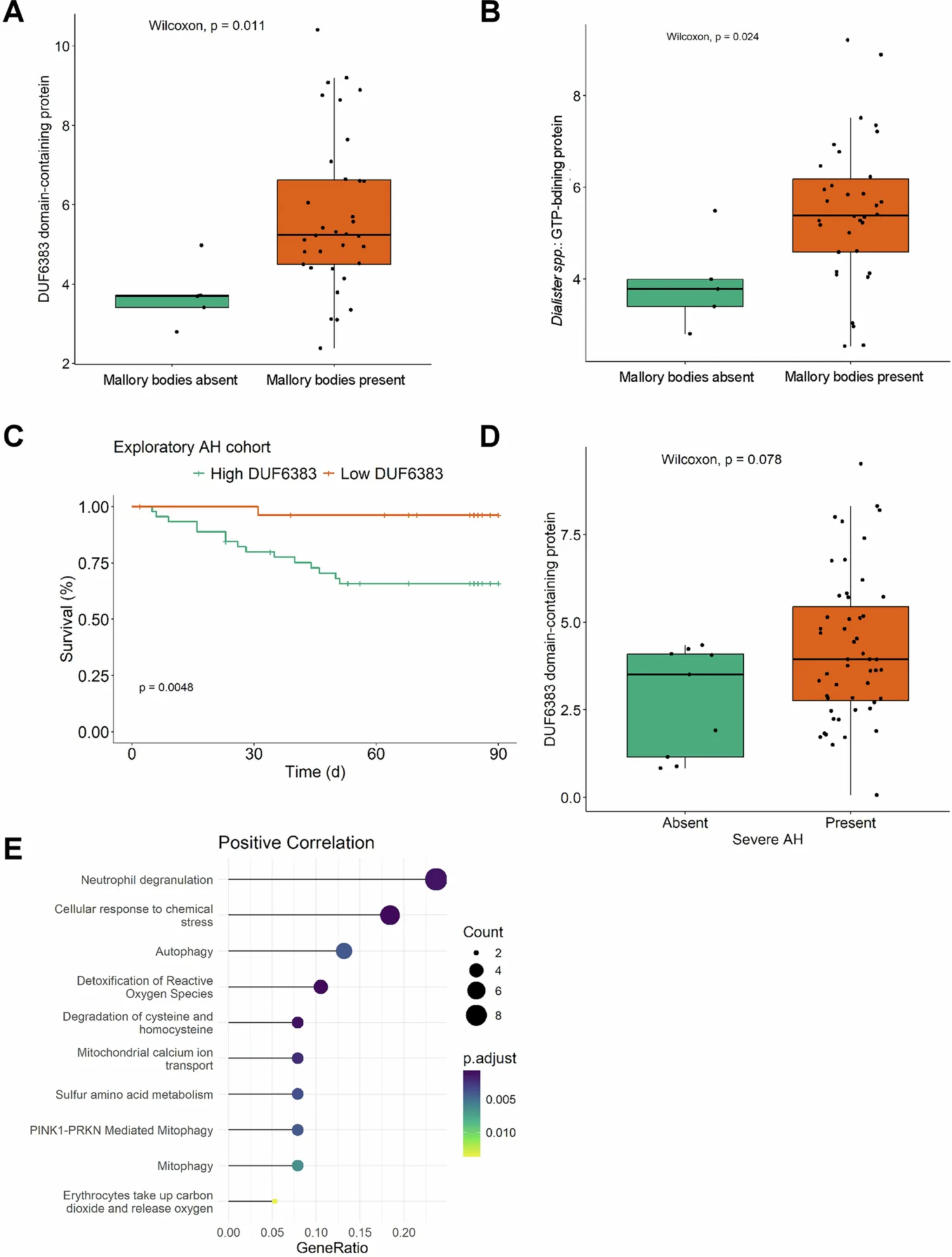
DUF6383 domain-containing protein from *P. distasonis* is associated with 90-day mortality and correlates with proteins of neutrophil degranulation in the feces. (A and B) Mallory bodies of patients with AH were divided into low and high groups according to the fecal DUF6383 domain-containing protein from *P. distasonis* ((A), *P* = 0.011) or GTP binding protein level from *Dialister spp.* ((B), *P* = 0.024). Optimal cut-off values (cut-off for DUF6383 domain-containing protein from *P. distasonis* = 3.325713; cut-off for GTP binding protein from *Dialister spp.* = 6.507031) were determined using maximally selected rank statistics (maxstat.test in the R package maxstat). *P*-values were calculated using Wilcoxon-Test. (C) Kaplan‒Meier curve of 90-day mortality for 72 patients with AH (exploratory InTeam cohort). Patients were grouped according to their fecal level of DUF6383 domain-containing protein from *P. distasonis.* The cut-off values for the two groups are described in the legend of Figure 2A. The date of liver transplantation was regarded as the death date for patients receiving transplantation (*n* = 3) and censored patients (*n* = 3 Log-rank *P* = 0.0048). (D) Abundance of DUF6383 domain-containing protein from *P.distasoni*s in patients with severe AH as defined by a Maddrey score ≥ 32 (*n* = 51). (E) Abundance of DUF6383 domain-containing proteins from *P. distasonis* was correlated with 680 human proteins in the feces, which we have described recently^21^ using Pearson correlation. Proteins that showed a positive correlation (D, R > 0.2, *P* < 0.05) were clustered using overrepresentation analysis and Reactome pathways. *P*-values were adjusted using false-discovery rate.

Taken together, several bacterial proteins with diverse and multifunctional (“moonlighting”) roles were found to be increased in patients with ALD, notably including phosphopyruvate hydratase (enolase) and glucose-6-phosphate isomerase (hexokinase), which have important functions in metabolism as well as host‒pathogen interactions. Among these, the DUF6383 domain-containing protein produced by *P. distasonis* can predict short-term mortality and might serve as a potential prognostic biomarker.

### Interactions between DUF6383 domain-containing protein and the host proteome

To investigate potential interactions between the DUF6383 domain-containing protein and the host proteome, we correlated its abundance with that of all 680 host proteins described by us previously.^21^ We identified 51 proteins showing a positive correlation (Pearson’s R > 0.2) and 78 proteins with a negative correlation (Pearson’s R < −0.2) to the DUF6383 domain-containing protein. Next, we performed an overrepresentation analysis of Reactome pathways to cluster the correlating proteins by function. Among positively correlated proteins, 25% belong to the “neutrophil degranulation” pathway. Other prominent pathways are “cellular response to chemical stress” and “autophagy” (Figure 2E). Notably, approximately 7.5% of these proteins are involved in mitophagy, mainly including the outer mitochondrial membrane proteins voltage-dependent anion channel (VDAC) 1–3. We recently found that intestinal neutrophil activation correlates with disease severity and mortality in patients with AH.^21^ To adjust for confounding factors, we performed multivariate cox regression. *P. distasonis* predicts short-term mortality independent of fecal myeloperoxidase (MPO), which is a hallmark enzyme of neutrophil granules and released upon activation (HR: 1.3, *p* = 0.018). Negatively correlated proteins were predominantly involved in glucose metabolism and muscle contraction pathways (Supplemental Figure 3).

These results point to the possible involvement of DUF6383 domain-containing protein in disease pathophysiology.

### Metaproteomic taxonomic shifts in alcohol-associated liver disease

To better understand how the metaproteomic profile changes between groups, we used taxonomic profiling. Controls and AUD patients showed a higher abundance of proteins derived from Firmicutes compared to patients with AH (Controls vs. AH *P*_*adj.*_ = 3.19e-8, AUD vs. AH *P*_*adj.*_ = 1.76e-4) (Figure 3A). Proteins from Bacteroidetes and Proteobacteria were more abundant in both patients with AUD and patients with AH (Bacteroidetes: Control vs. AH *P*_*adj.*_ = 6.75e-6, Control vs. AUD *P*_*adj.*_ *=1.91e-10,* AUD vs. AH *P*_*adj.*_ = 7.72e-4, Proteobacteria: Control vs. AH *P*_*adj.*_ = 7.56e-28, Control vs. AUD *P*_*adj.*_ *=4.9e-9,* AUD vs. AH *P*_*adj.*_ = 1.53e-3) (Figure 3A). Patients with AUD showed a decreased abundance of proteins from Actinobacteria relative to controls and patients with AH (Controls vs. AUD *P*_*adj.*_ = 1.65e-2, AUD vs. AH *P*_*adj.*_ = 7.72e-4) (Figure 3A). At the genus level, proteins from *Faecalibacterium* were decreased in patients with AH compared with controls (*P*_*adj.*_ *=*0.0314) while those from *Bacteroides* were elevated in patients with AUD and patients with AH compared with controls (Control vs. AUD *P*_*adj.*_ < 0.001, Control vs. AH *P*_*adj.*_ < 0.001) (Figure 3B). Proteins from *Prevotella* were slightly increased in patients with AUD (*P*_*adj.*_ = 0.007), but there was no significant difference between patients with AH compared to controls (Figure 3B). Additionally, proteins from *Roseburia* and *Ruminococcus* were diminished in patients with AUD and patients with AH (*Roseburia P*_*adj.*_ = 0.024*, Ruminococcus P*_*adj.*_ *=*0.0042) (Figure 3B). On the species level, proteins of *Bacteroides dorei* and *vulgatus* were increased in patients with AH compared with controls (*B. dorei P*_*adj.*_ = 0.036, *B. vulgatus P*_*adj.*_ = 0.036) (Figure 3C).

**Figure 3.**
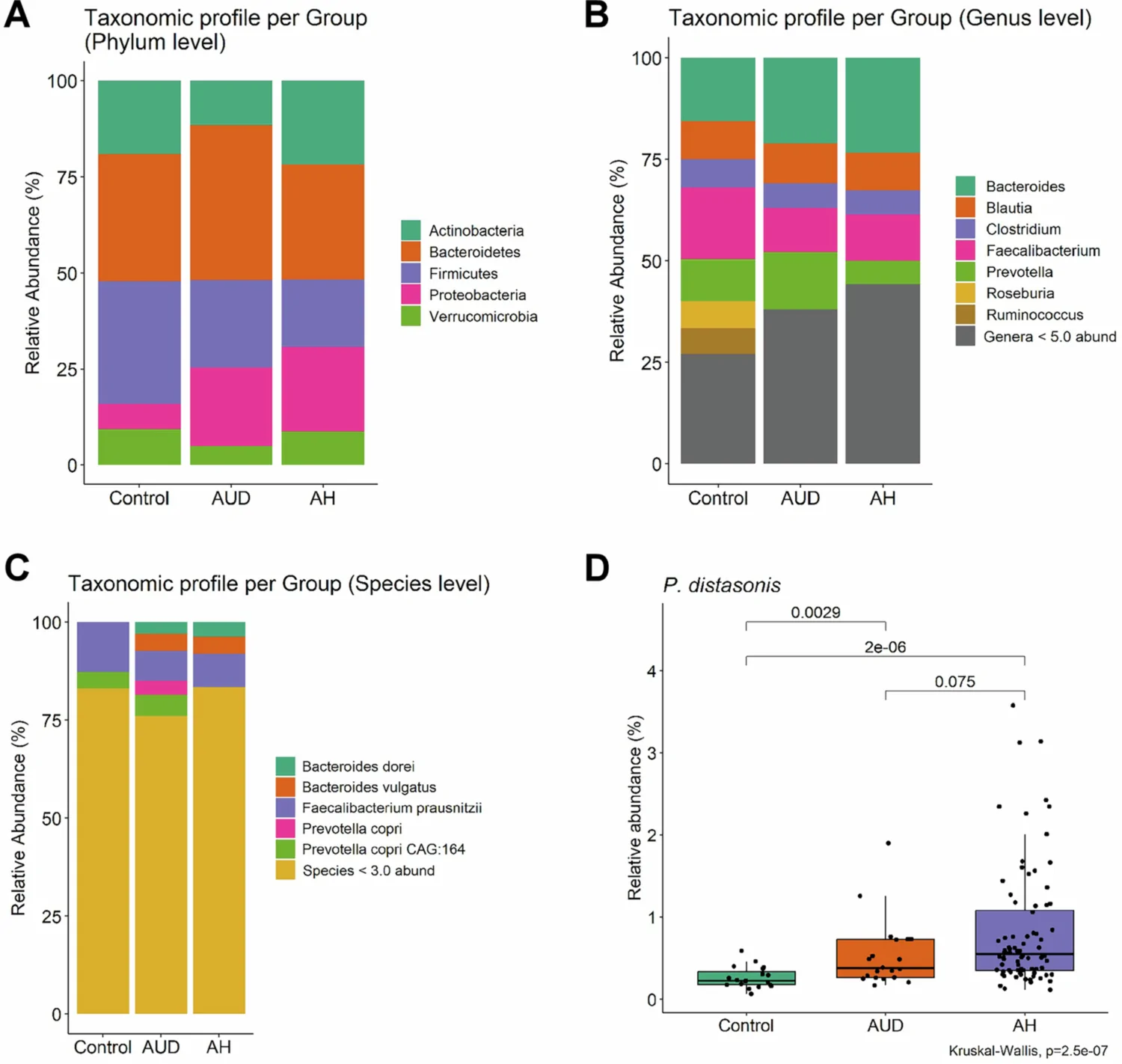
Taxonomic profiles are different between patients with AH, AUD, and controls. (A–C) The relative abundance of each bacterium was calculated based on the bacterial protein data. (A) Phylum level. Actinobacteria *P* = 0.00156, Bacteroidetes *P* = 8.65e-11, Firmicutes *P* = 8.79e-10, Proteobacteria *P* = 5.59e-28, Verrucomicrobia *P* = 0.13. (B) Genus level. Significance *Bacteroides P =* 1.75 e-6, *Blautia P* = 0.0781, *Clostridium P =* 0.000187, *Faecalibacterium P =* 0.0396, *Prevotella P =* 3.57e-9, *Roseburia P =* 0.0274. (C) Species level. *Bacteroides dorei P =* 0.0106, *Bacteroides vulgatus P =* 0.0239, *Faecalibacterium prausnitzii P =* 0.00466, *Prevotella copri P =* 0.359, *Prevotella copri CAG:164 P =* 0.485. (D) Relative abundance of *P. distasonis*. Significance was calculated using Kruskal–Wallis and Wilcoxon rank-sum test.

We calculated the relative abundance of *P. distasonis* proteins from the metaproteomics dataset. Patients with AH showed significantly higher relative abundance of *P. distasonis* proteins compared with controls and patients with AUD (*p* < 0.001 and *p* = 0.12, respectively) (Figure 3D). In summary, our metaproteomic data confirm and complement metagenomic observations by showing not only the presence of microbial taxa but also their changes in protein expression.

### Differential impact of Firmicutes and Proteobacteria-derived proteins on mortality in patients with AH

The association between mortality in patients with AH and specific taxa is well described.^35^ ^,^ ^36^ For example, *Enterococcus faecalis* produces cytolysin, which is associated with worse survival.^18^ In addition to taxonomic profiling, we aimed to identify which microbial proteins correlate with patient mortality. Our unbiased analysis revealed that proteins from distinct bacterial taxa are significantly associated with 30- and 90-day mortality. Proteins expressed by Firmicutes, including various *Ruminococcus* and *Faecalibacterium* species, predominantly correlate with 30-day mortality, with a smaller contribution from proteins of Bacteroides and Proteobacteria (Figure 4A, Supplemental Table 2). In contrast, 90-day mortality correlates mainly with proteins from Proteobacteria, particularly pathobiontic genera such as *Enterobacter*, *Citrobacter*, *Klebsiella*, *Escherichia coli*, and *Morganella* (Figure 4B, Supplemental Table 3). Additionally, Firmicutes genera, including *Ruminococcus*, *Oscillibacter*, *Holdemania*, and *Faecalibacterium,* also contribute to 90-day mortality, albeit to a lesser extent compared to 30-day mortality (Figure 4B, Supplemental Table 3). Notably, proteins derived from *Parabacteroides* were linked to survival outcomes at both 30- and 90-days (Figure 4A, B; Supplemental Tables 2 and 3). Only Firmicutes and Bacteroidetes correlate negatively with 30- and 90-day survival, while Proteobacteria did not seem to have an effect (Figure 4C, D; Supplemental Tables 4 and 5). Taken together, these findings indicate that mortality at day 30 and day 90 is associated with different bacterial proteins, but proteins from *Parabacteroides* seem to play an important role in 30-day and 90-day mortality.

**Figure 4.**
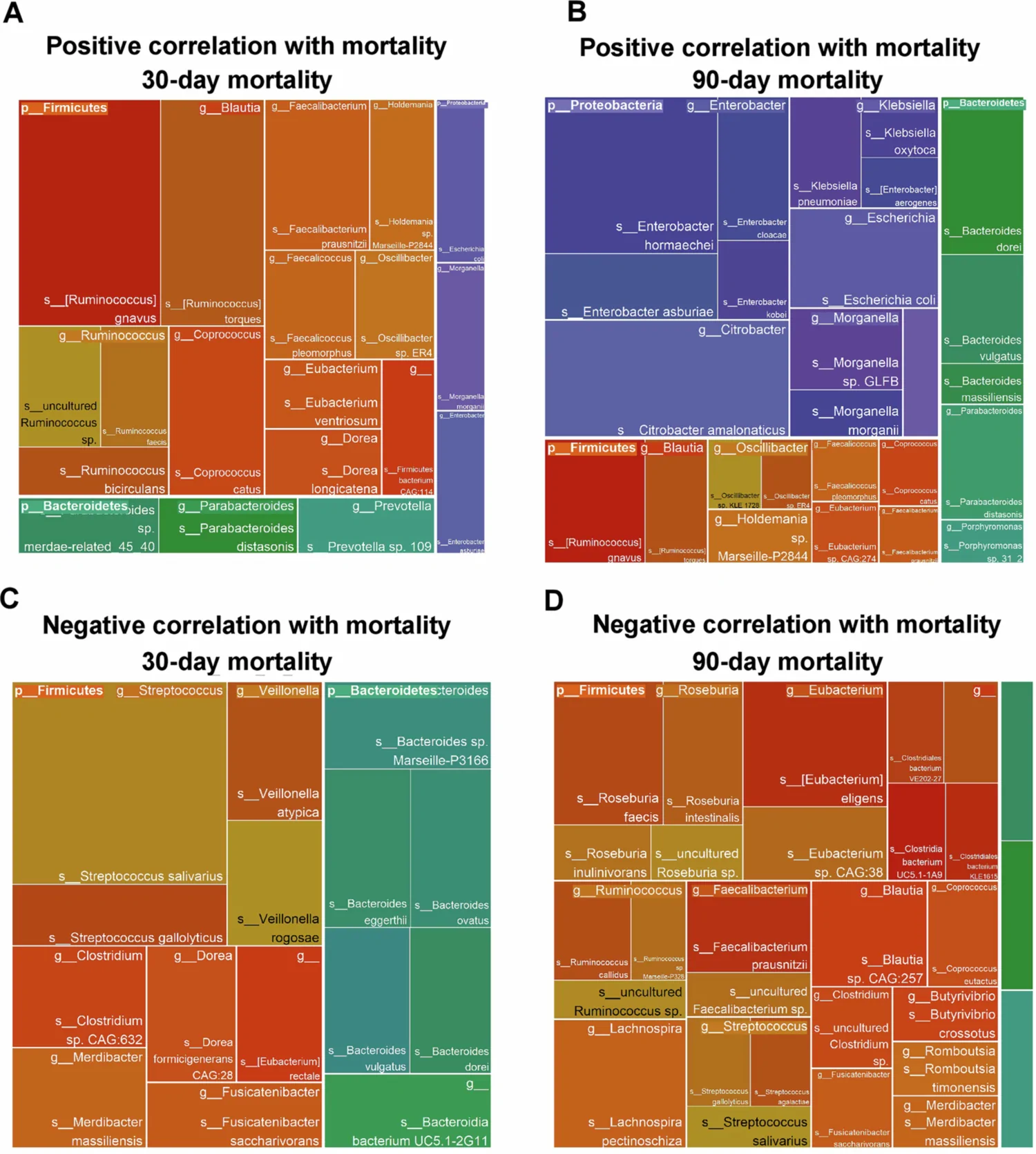
Bacterial proteins correlating with 30- and 90-day survival. (A–D) All bacterial proteins were correlated with survival using Spearman correlation. Proteins were selected for further analysis if R > 0.2 or R < −0.2, respectively. Bacterial proteins were ordered according to phyla, genera, and species, with each color group representing one phylum and each section representing one species. The size of the rectangles is larger if the correlation coefficient is higher for positive correlation and smaller for negative correlation. Firmicutes = orange, Bacteroides = green, and Proteobacteria = blue. The size of the rectangles is larger if the correlation coefficient is higher for positive correlation and smaller for negative correlation.

### Validation of DUF6383 domain-containing protein as a marker for disease severity

Since the levels of DUF6383 domain-containing protein increased with disease severity across the different stages, and a significant correlation was found with survival of patients with AH, we validated the correlation between DUF6383 domain-containing proteins and survival in an independent cohort. We used 72 samples from patients with AH enrolled in a multicenter cohort through the AlcHepNet consortium (Table 2), isolated DNA from fecal samples, and performed qPCR with primers specifically recognizing the genomic region of DUF6383 domain-containing protein encoded by *P. distasonis*. We divided the group into patients with high and low DUF6383 domain-containing protein levels using max-rank selected statistics. We confirmed that patients with high amplification of DUF6383 domain-containing protein had lower 90-day survival (log rank, *p* = 0.045) (Figure 5A). There was no difference in the MELD score between the two groups, and the genomic DNA of DUF6383 domain-containing proteins was not significantly different in patients with severe vs. non-severe AH (*p* = 0.85, Figure 5B).

**Table 2.** Clinical characteristics of patients with AH in the validation cohort (AlcHepNet).

Variable	Alcohol-associated hepatitis *n* = 72
**Clinical parameters**	
Age (years), *n* = 72	45.3 (22.2–71.5)
Sex (male) (%), *n* = 72	49 (68.05%)
Weight (kg), *n* = 71	86 (50.3–135)
Height (m), *n* = 67	1.76 (1.53–1.93)
BMI (kg/m2), *n* = 63	28.2 (19–45.7)
**Laboratory parameters**	
Albumin (g/dl), *n* = 71	2.81 (1.4–3.9)
ALP (IU/L), *n* = 71	191 (44–1008)
ALT (IU/L), *n* = 71	56 (5–289)
AST (IU/L), *n* = 71	140 (41–689)
Bilirubin (mg/dl), *n* = 69	20 (2.9–51.7)
Creatinine (mg/dl), *n* = 71	1.55 (0.3–9.2)
INR, *n* = 71	2 (0.9–5.34)
Cholesterol (mg/dl), *n* = 18	132 (29–288)
Triglycerides (mg/dl), *n* = 18	174 (14–670)
**Clinical presentation and treatment**	
Steroids, *n* (%), *n* = 22	13 (59%)
Antibiotics, *n* (%), *n* = 22	13 (59%)
**Clinical scores and outcome**	
MELD *n* = 72	27 (13–53)
Child‐Pugh stage A/B/C, *n* (%), *n* = 68	2 (2.9%)/24 (34.29%)/42 (60.0%)
30-day-mortality, *n* (%), *n* = 72	11 (15.2%)
90-day-mortality, *n* (%), *n* = 72	15 (20.8%)

Values are presented as mean and range in parentheses. The number of patients for whom data was available is indicated in the first column. BMI, body mass index; ALP, Alkaline phosphatase; ALT, alanine aminotransferase; AST, aspartate aminotransferase; GGT, gamma-glutamyl-transferase; INR, international normalized ratio; MELD, model of end-stage liver disease.

**Figure 5.**
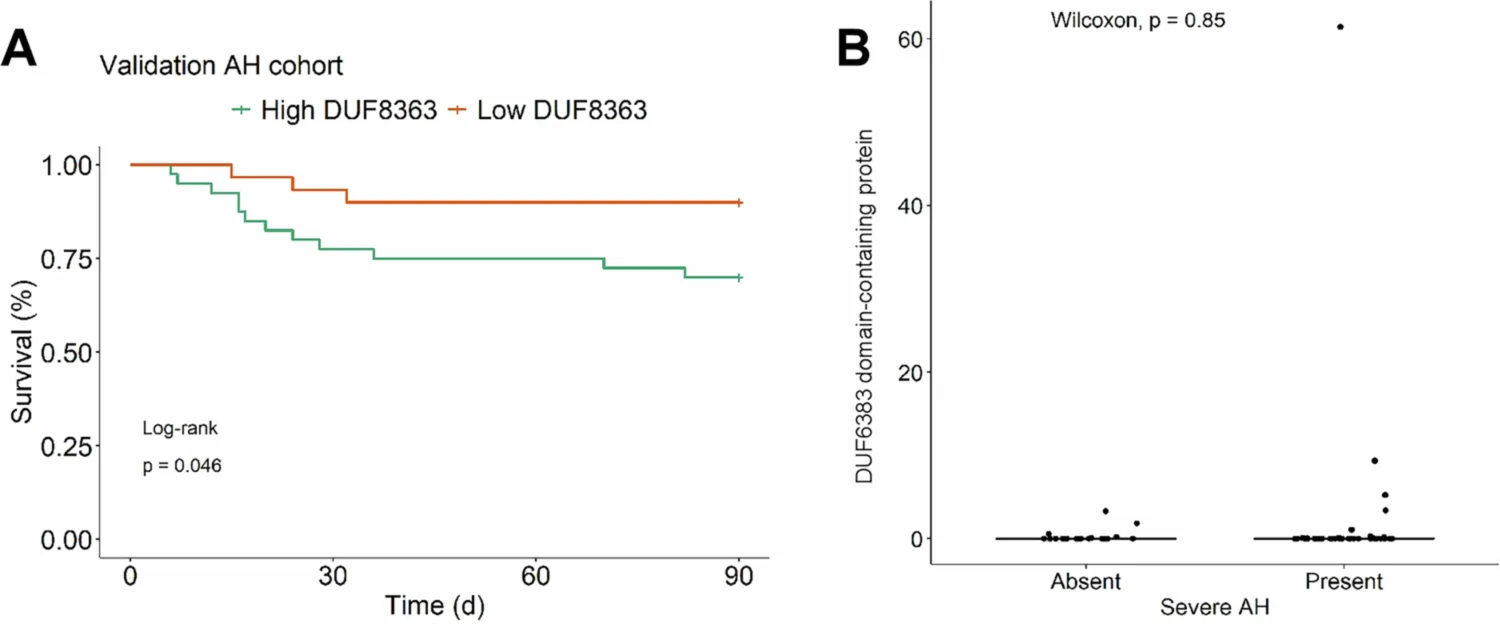
Genomic expression of DUF6383 domain-containing protein from *P. distasonis* is associated with 90-day survival. (A) Fecal genomic content of DUF6383 domain-containing protein from *P. distasonis* was measured in the feces by qPCR and normalized to 16 s in 72 patients with AH. An optimal cut-off of 0.000273029 was calculated using maximally selected rank statistics (maxstat.test in R) to divide the cohort into two groups with low and high fecal DUF6383 domain-containing protein from *P. distasonis*. Kaplan–Meier curve of 90-day mortality for patients with AH (log rank *P* = 0.046). (B) Genomic DNA abundance of DUF6383 domain-containing proteins in patients with severe AH as defined by a Maddrey score ≥ 32 (*n* = 47).

## Discussion

Metaproteomic analysis of fecal samples from controls, patients with AUD and AH revealed differences in microbial protein composition and function. AUD and AH were characterized by a loss of Firmicutes-derived proteins and enrichment of proteins from Bacteroidetes, Proteobacteria, and Actinobacteria, accompanied by shifts in specific genera. Eighteen bacterial proteins increased with disease severity, with some showing multifunctional roles in both metabolism and host–microbe interactions, including enolase and hexokinase. The DUF6383 domain-containing protein from *P. distasonis* correlates with the histological features of AH and predicts survival at day 90. These findings were validated on a genomic level in an independent cohort. DUF6383 domain-containing protein is linked to the host pathway of neutrophil degranulation. Mortality patterns differed by taxonomic origin, with Firmicutes proteins more closely tied to 30-day outcomes and Proteobacteria proteins dominating 90-day risk.

When comparing metaproteomes between controls, patients with AUD and patients with AH, proteins produced by *Veillonella* show distinct differences. This is consistent with previously published data from the same cohort, where *Veillonella* abundance was significantly associated with higher bilirubin levels, hence more aggressive disease, in patients with AH.^20^

Some of the identified proteins are known to have “moonlighting” functions. Their function is first described in one bacterial species and later identified in others.^37^ The same protein may have different functions depending on the species that produces it.^37^ Many moonlighting proteins bind to the bacterial surface via ionic interactions and can be released under specific conditions, such as increased pH.^38-42^ Although no data exist on the fecal pH level in patients with AH, studies in cirrhosis and critically ill patients have reported a rise in pH towards alkaline conditions.^43^ ^,^ ^44^ For example, bifidobacterial enolase preferentially activates human plasminogen, a modulator of inflammation and adhesion.^45-47^ Thus, the interplay between microbial protein moonlighting, environmental conditions such as pH, and host–microbe interactions may be important for the pathophysiology in AH.

Several proteins in our dataset remain functionally uncharacterized. The DUF6383 domain-containing protein is mainly found at the C-terminus of uncharacterized proteins and shows similarity to the Type IX secretion system (T9SS) targeting domain.^48^ T9SS is expressed solely by Bacteroidetes and secretes virulence factors such as those from *Porphyromonas gingivalis.* Proteins that are transported across the outer membrane are tagged by a conserved C-terminal domain, which directs them to the outer membrane location. The tag is then cleaved by a protease and replaced by LPS.^49^ It is possible that DUF6383 domain-containing protein might function similarly, being associated with proteins transported across the outer membrane and cleaved off during the process. DUF6383 domain-containing protein appears to be expressed only in the order of Bacteroidales, with 246 of the 1,374 known sequences assigned to *P. distasonis.* ^50^ Given that *P. distasonis* belongs to the order of Bacteroidales known to frequently encode the T9SS machinery, it is plausible that it may possess this or another secretion system. However, further experimental validation is required for confirmation.

*P. distasonis* resides under anaerobic conditions in the distal colon. Current evidence shows that the beneficial and pathogenic effects of *P. distasonis* in murine colitis models. Oral gavage of *P. distasonis* reduces inflammation in dextran sulfate sodium (DSS)-induced colitis by suppressing TLR4 signaling in mice and restores the intestinal barrier in mice treated with azoxymethane.^51^ ^,^ ^52^ *P.* *distasonis* stimulates CD4+ T-cells to differentiate into regulatory T-cells in wild-type mice and attenuates inflammation *in vitro.* ^53^ ^,^ ^54^ In contrast, in another study, *P. distasonis* enhanced DSS-induced colitis in mice after oral gavage.^55^ Human studies are scarce. *P. distasonis* was found in stool of patients with Crohn’s disease and in the deep-gut wall tissues from patients who underwent resection of the inflamed gut.^56^ ^,^ ^57^ Alongside nine other anaerobic bacteria cultured from the small intestinal mucosa, it was associated with Th1 and Th17 cell accumulation and intestinal inflammation in patients with Crohn’s disease.^58^ In patients with ulcerative colitis, fecal *P. distasonis* levels were negatively correlated with the duration of remission.^59^ Overall, there seem to be protective and anti-inflammatory effects in mice, whereas associations with inflammatory effects appear to be more prominent in humans. We were unable to find any studies evaluating the potential role of *P. distasonis* in ALD. In our cohort, DUF6383 domain-containing protein from *P. distasonis* was associated with reduced survival, although a higher taxonomic abundance of *P. distasonis* showed a trend, but did not reach statistical significance.

Strengths of our study included the use of two independent multicenter cohorts, supporting the robustness of our findings. Nonetheless, the sample size remains limited. In addition, the correlation between the DUF6383 domain-containing protein and survival was validated at the genomic level rather than at the protein level. Furthermore, our approach favors the detection of abundant proteins, such as enzymes involved in energy metabolism and structural components, versus proteins, which are low abundant such as transcription factors or even toxins. Future studies are required to assess the mechanistic role of DUF6383 domain-containing protein from *P. distasonis*.

Taken together, metaproteomic analysis revealed distinct microbial signatures associated with AUD and AH, with DUF6383 domain-containing protein from *P. distasonis* emerging as a predictor of short-term mortality. The biological role of DUF6383 domain-containing protein remains unknown and warrants further investigation.

### Roster for authorship, AlcHepNet clinical center investigators

**Indiana University School of Medicine, Indianapolis, IN:** Naga Chalasani, MD; Lauren Nephew, MD; Kavish R. Patidar, DO (currently at Baylor College of Medicine); Raj Vuppalanchi, MD; Niha Samala, MD; Lindsey Yoder, PA; Suthat Liangpunsakul, MD, MPH, Shawna Prange, RN.

**Mayo Clinic, Rochester, Minnesota:** Vijay H. Shah, MD; Douglas A. Simonetto, MD; Patrick Kamath, MD; Hugo E. Vargas, MD (Mayo Clinic, Scottsdale); Liu Yang, MBBS (Mayo Clinic, Jacksonville)

**Cleveland Clinic, Cleveland, Ohio:** Srinivasan Dasarathy, MD; Nicole Welch, MD; Annette Bellar; Amy Attaway, MD; Jaividhya Dasarathy, MD (MetroHealth, Cleveland, Ohio); Ashley Growley; David Streem, MD; Laura E. Nagy, PhD

**University of Texas Southwestern, Dallas, Texas:** Mack C. Mitchell, MD; H. Franklin Herlong, MD; Thomas Kerr, MD; Thomas Cotter, MD

**Virginia Commonwealth University, Richmond, Virginia**: Arun Sanyal, MBBS; Sara O'Connor, RN; Velimir Luketic, MD; Amon Asgharpour, MD; Stephanie Taylor, RN

**University of Louisville, Louisville, Kentucky:** Craig J. McClain, MD; Vatsalya Vatsalya, MD, PhD; Loretta Jophlin, MD, PhD; Matt Cave, MD; Suman Kumar Jha, MD; Luis Marsano, MD; Ashutosh Barve, MD, PhD; Jane Frimodig, PhD

**University of Pittsburgh, Pittsburgh, Pennsylvania:** Ramon Bataller, MD (currently at Universitat de Barcelona, Barcelona, Spain) Samhita Ravi; Jaideep Behari, MD; Sharvari Shivanekar, MD; Paula Novelli, MD; Andres Duarte-Rojo, MD (currently at Northwestern University, Chicago, IL), Naudia Jonassaint, MD

**Beth Israel Deaconess Medical Center, Boston, Massachusetts:** Gyongyi Szabo, MD, PhD; Michael Curry, MD; Zhenghui G Jiang, MD; Ushma Agarwal; Hannah Siegenberg, Mia Hazel

**University of California at San Diego:** Bernd Schnabl, MD

**NIAAA Project Scientist:** Svetlana Radaeva, PhD

**Data Coordinating Center at Indiana University:** Wanzhu Tu, PhD; Samer Gawrieh, MD; Qing Ting, MS; Carla Kettler, MS; Jing Su, PhD; Yang Li, PhD; Savannah Yarnelle; Yunpeng Yu, MS

**Data Coordinating Center at University of Massachusetts:** Bruce A. Barton, PhD.

## Supplementary Material

Supplemental Material Supplemental_Material_no_underlines_25_Sep_2026_21_01_AU.docx

Supplementary Material Supplemental Figures.zip

## Data Availability

Data are available in a public, open-access repository. All .RAW data and .MZML data are deposited at massive. ucsd. edu, study ID: MassIVE MSV000094610.

## Abbreviations

AH: Alcohol-associated hepatitis
ALD: alcohol-associated liver disease
AUD: alcohol use disorder
ALT: alanine aminotransferase
AST: aspartate aminotransferase
*B. finegoldii*: *Bacteroides finegoldii*
*B. thetaiotaomicron*: *Bacteroides thetaiotaomicron*
*B. dorei*: Bacteroides dorei
*B. vulgatus*: Bacteroides vulgatus
*Bif. dentium*: Bifidobacterium dentium
*Bif. bifidium*: Bifidobacterium bifidium
*C. bolteae*: *Clostridium bolteae* CAG:59
*E. feacium*: Enterococcus faecium
*Eu. dolichum*: *Eubacterium dolichum*
INR: international normalized ratio
MELD: Model for End-Stage Liver Disease
MAMPs: microbial-associated molecular patterns
NLRP3: NLR family pyrin domain containing 3
*P. distasonis*: *Parabacteroides distasonis*
*R.inulinivorans*: Roseburia inulinivorans
*R. gnavus*: *Ruminococcus gnavus*
TMT: Tandem mass tag
*V. seminalis*: *Veillonella seminalis*
*V. atypica*: *Veillonella atypica*
*V. parvula*: Veillonella parvula
VDAC: voltage-dependent anion channel
