# Supplementary material for: DUF6383-domain containing protein from Parabacteroides distasonis predicts survival in patients with alcohol-associated hepatitis
Source: Gut Microbes Rep. 2026 Oct 28;4(1):2728306. doi: 10.1080/29933935.2026.2728306 (PMC13733255; doi:10.1080/29933935.2026.2728306)
Supplement: Supplementary file 2 [file KGMR_A_2728306_SM7616.docx]

**Supplemental Material**

**Supplemental Figure 1. Bacterial protein patterns are not different in the feces from patients with steroids, antibiotics or infections**

Characterization of bacterial proteins was performed from fecal samples of patients with alcohol-associated hepatitis (AH, InTeam) receiving steroids at the time of stool collection (**A**, n=77), receiving antibiotics at the time of stool collection (**B**, n=77), or the occurrence of infections (**C,** n=58). For the group “Antibiotics” in panel B not enough data points were available to calculate an ellipse. *P*-values were calculated using the dissimilarity index calculated by the method of Bray-Curtis and Permutational Multivariate Analysis of Variance Using Distance Matrices

**Supplemental Figure 2.** Abundance of DUF6383 domain–containing protein in patients receiving steroid (A) or antibiotics (B) treatment at the time point of stool collection. DUF-6383 domain-containing protein was adjusted for the MELD Score. *P*-values were calculated using ANCOVA.

**Supplemental Figure 3.** Abundance of DUF6383 domain–containing protein from *P. distasonis* was correlated with 680 human proteins in the feces which we have described recently (21) using pearson correlation. Proteins which showed a negative correlation (R < -0.2, P < 0.05), were clustered using overrepresentation analysis and Reactome pathways. *P*-values were adjusted using false-discovery rate

**Supplemental Table 1.** Protein abundance of the 18 detected proteins which showed an increase with disease severity and a significant difference between all three groups

|  | ***P*-Value Kruskal Wallis test** | ***P*-Value Wilcoxon rank sum test** |
| --- | --- | --- |
| *E. faecium*: phosphopyruvate hydratase | 3.24e- 8 | 0.0142 |
| *Bif. bifidum*: phosphopyruvate hydratase | 1.16e- 3 | 0.0302 |
| *C. bolteae*: argininosuccinate lyase | 5.91e- 3 | 0.0112 |
| *B. finegoldii*: trigger factor | 2.02e- 7 | 0.0366 |
| *V. parvula*: PepSY domain-containing protein | 9.15e- 8 | 0.000300 |
| *V. parvula*: hypothetical protein | 2.24e-10 | 0.0289 |
| *V. seminalis*: methylmalonyl-CoA mutase family protein | 9.48e- 6 | 0.000272 |
| *B. thetaiotaomicron*: NigD-like protein | 1.04e- 9 | 0.0237 |
| V*. parvula*: S-layer homology domain-containing protein | 7.26e-12 | 0.00118 |
| *Bif. dentium*: glucose-6-phosphate isomerase | 3.11e-12 | 0.00638 |
| *Dialister* spp: GTP-binding protein | 2.96e-12 | 0.00590 |
| *Faecalibacterium* spp.: m18 family aminopeptidase | 1.26e-12 | 1.26e-12 |
| *R. gnavus*: glycoside hydrolase N-terminal domain-containing protein | 5.56e- 7 | 0.0499 |
| *Roseb.inulinivorans*: flagellin | 5.35e- 8 | 0.0289 |
| uncultured *Clostridium* sp.: UvrD-helicase domain-containing protein | 6.26e-10 | 0.00184 |
| *P. distasonis*: DUF6383 domain-containing protein | 2.36e- 3 | 0.0129 |
| *V. atypica*: Ldh family oxidoreductase, partial | 2.32e- 4 | 0.00283 |
| *Eu. dolichum*: NADP-specific glutamate dehydrogenase | 1.57e- 2 | 0.00986 |

Significance of the 18 detected proteins which showed an increase with disease severity and a significant difference between all three groups as calculated by Kruskal-Wallis test, and a significant difference between AH and AUD patients following Wilcoxon rank sum test. *Bif. dentium, Bifidobacterium dentium*; *B. finegoldii, Bacteroides finegoldii; B. thetaiotaomicron, Bacteroides thetaiotaomicron; Bif. bifidum, Bifidobacterium bifidum; C. bolteae, Clostridium bolteae; E. faecium, Enterococcus faecium; Eu. dolichum, Eubacterium dolichum; P. distasonis, Parabacteroides distasonis; R. gnavus, Ruminoccous gnavus; Roseb. inulinivorans, Roseburia inulinivorans; V. atypica, Veillonella atypica; V. parvula, Veillonella parvula; V. seminalis, Veillonella seminalis.*

**Supplemental Table 2.** Positive correlation of bacterial proteins with 30-day mortality (data for Figure 4A). Correlation coefficient (R) was calculated using spearman correlation. Proteins were selected for further analysis if R>0.2.

| **Protein name** | ***R*** | ***P-*value** | **Phylum** | **Genus** | **Species** |
| --- | --- | --- | --- | --- | --- |
| acyl-CoA dehydrogenase | 0.29 | 0.01 | Firmicutes | Coprococcus | Coprococcus catus |
| hypothetical protein | 0.29 | 0.02 | Bacteroidetes | Bacteroides | Bacteroides sp. UNK.MGS-14 |
| sugar ABC transporter ATP-binding protein | 0.24 | 0.05 | Firmicutes | Blautia | [Ruminococcus] gnavus |
| phosphopyruvate hydratase | 0.29 | 0.01 | Firmicutes | Holdemania | Holdemania sp. Marseille-P2844 |
| sn-glycerol-3-phosphate ABC transporter ATP-binding protein UgpC, partial | 0.27 | 0.02 | Firmicutes | Ruminococcus | Ruminococcus bicirculans |
| aminotransferase class III-fold pyridoxal phosphate-dependent enzyme | 0.24 | 0.04 | Firmicutes |  | Firmicutes bacterium CAG:114 |
| ABC transporter substrate-binding protein | 0.27 | 0.02 | Firmicutes | Romboutsia | Romboutsia timonensis |
| hypothetical protein | 0.24 | 0.04 | Firmicutes | Blautia | Blautia wexlerae |
| phosphoglycerate kinase, partial | 0.24 | 0.04 | Proteobacteria | Enterobacter | Enterobacter cloacae |
| 50S ribosomal protein L15, partial | 0.24 | 0.05 | Proteobacteria | Escherichia | Escherichia coli |
| MULTISPECIES: 50S ribosomal protein L4 | 0.24 | 0.05 | Proteobacteria | Morganella | Morganella sp. GLFB |
| MULTISPECIES: type I glyceraldehyde-3-phosphate dehydrogenase | 0.26 | 0.03 | Firmicutes | Blautia | [Ruminococcus] torques |
| pyruvate:ferredoxin (flavodoxin) oxidoreductase | 0.24 | 0.05 | Firmicutes | Coprococcus | Coprococcus catus |
| ABC transporter substrate-binding protein | 0.28 | 0.02 | Firmicutes |  | Clostridiales bacterium 42_27 |
| extracellular solute-binding protein | 0.27 | 0.02 | Firmicutes | Ruminococcus | uncultured Ruminococcus sp. |
| triosephosphate isomerase | 0.25 | 0.04 | Bacteroidetes | Prevotella | Prevotella sp. 109 |
| DUF6383 domain-containing protein | 0.28 | 0.02 | Bacteroidetes | Parabacteroides | Parabacteroides sp. merdae-related_45_40 |
| pyruvate:ferredoxin (flavodoxin) oxidoreductase | 0.30 | 0.01 | Firmicutes | Blautia | [Ruminococcus] gnavus |
| urocanate hydratase | 0.25 | 0.04 | Bacteroidetes | Alistipes | Alistipes putredinis |
| MAG: flagellar hook protein | 0.24 | 0.04 | Firmicutes | Eubacterium | Eubacterium sp. 41_20 |
| 50S ribosomal protein L5 | 0.26 | 0.03 | Firmicutes | Dorea | Dorea longicatena |
| S-layer homology domain-containing protein | 0.24 | 0.04 | Firmicutes | Oscillibacter | Oscillibacter sp. KLE 1728 |
| MULTISPECIES: DUF6383 domain-containing protein | 0.30 | 0.01 | Bacteroidetes | Parabacteroides | Parabacteroides distasonis |
| acetyl-CoA C-acetyltransferase, partial | 0.25 | 0.04 | Firmicutes | Oscillibacter | uncultured Oscillibacter sp. |
| uncharacterized protein BN675_02844 | 0.27 | 0.02 | Bacteroidetes | Parabacteroides | Parabacteroides sp. merdae-related_45_40 |
| hypothetical protein, partial | 0.25 | 0.04 | Bacteroidetes | Alistipes | Alistipes putredinis |
| hypothetical protein | 0.24 | 0.04 | Firmicutes | Faecalibacterium | Faecalibacterium prausnitzii |
| molecular chaperone DnaK | 0.24 | 0.04 | Firmicutes | Eubacterium | Eubacterium rectale CAG:36 |
| PTS system mannose/fructose/sorbose family transporter subunit IID | 0.33 | 0.00 | Firmicutes | Faecalicoccus | Faecalicoccus pleomorphus |

**Supplemental Table 3.** Negative correlation of bacterial proteins with 30-day mortality (data for Figure 4B). Correlation coefficient (R) was calculated using spearman correlation. Proteins were selected for further analysis if R<-0.2.

| **Protein name** | ***R*** | ***P-*value** | **Phylum** | **Genus** | **Species** |
| --- | --- | --- | --- | --- | --- |
| phosphoenolpyruvate carboxykinase (ATP), partial | -0.25 | 0.04 | Bacteroidetes |  | Bacteroidia bacterium UC5.1-2G11 |
| MULTISPECIES: 30S ribosomal protein S10 | -0.26 | 0.03 | Firmicutes | Streptococcus | Streptococcus agalactiae |
| type I glyceraldehyde-3-phosphate dehydrogenase | -0.26 | 0.03 | Firmicutes | Streptococcus | Streptococcus salivarius |
| polyribonucleotide nucleotidyltransferase | -0.25 | 0.04 | Bacteroidetes | Bacteroides | Bacteroides dorei |
| - | -0.25 | 0.03 | Bacteroidetes | Bacteroides | Bacteroides vulgatus |
| fructose-bisphosphate aldolase | -0.36 | 0.00 | Firmicutes | Streptococcus | Streptococcus salivarius |
| glutamine synthetase III | -0.28 | 0.02 | Firmicutes | Roseburia | Roseburia faecis |
| phosphopyruvate hydratase | -0.27 | 0.02 | Firmicutes | Merdibacter | Merdibacter massiliensis |
| MULTISPECIES: diaminopimelate dehydrogenase | -0.25 | 0.04 | Bacteroidetes | Bacteroides | Bacteroides eggerthii |
| phosphoglycerate kinase | -0.30 | 0.01 | Bacteroidetes | Bacteroides | Bacteroides sp. Marseille-P3166 |
| type I glyceraldehyde-3-phosphate dehydrogenase | -0.32 | 0.01 | Firmicutes | Clostridium | Clostridium sp. CAG:632 |
| triose-phosphate isomerase | -0.27 | 0.02 | Firmicutes | Streptococcus | Streptococcus salivarius |
| m18 family aminopeptidase | -0.25 | 0.04 |  |  |  |
| 50S ribosomal protein L1 | -0.27 | 0.02 | Firmicutes |  | [Eubacterium] rectale |
| MULTISPECIES: DUF4295 domain-containing protein | -0.32 | 0.01 |  |  |  |
| triose-phosphate isomerase | -0.25 | 0.04 | Firmicutes | Fusicatenibacter | Fusicatenibacter saccharivorans |
| MULTISPECIES: type I glyceraldehyde-3-phosphate dehydrogenase | -0.25 | 0.04 | Firmicutes | Streptococcus | Streptococcus gallolyticus |
| methylmalonyl-CoA mutase small subunit | -0.25 | 0.04 | Bacteroidetes | Bacteroides | Bacteroides ovatus |
| Ldh family oxidoreductase, partial | -0.27 | 0.02 | Firmicutes | Veillonella | Veillonella atypica |
| carbohydrate ABC transporter substrate-binding protein | -0.24 | 0.04 | Firmicutes | Faecalibacterium | uncultured Faecalibacterium sp. |
| MULTISPECIES: 30S ribosomal protein S7 | -0.25 | 0.04 |  |  |  |
| putative porin, partial | -0.25 | 0.03 | Firmicutes | Veillonella | Veillonella rogosae |
| pyruvate-flavodoxin oxidoreductase | -0.24 | 0.04 | Firmicutes | Lachnospira | Lachnospira pectinoschiza |
| phosphoglycerate kinase | -0.25 | 0.04 |  |  |  |

**Supplemental Table 4.** Positive correlation of bacterial proteins with 90-day mortality (data for Figure 4C). Correlation coefficient (R) was calculated using spearman correlation. Proteins were selected for further analysis if R>0.2.

| **Protein name** | ***R*** | ***P-*value** | **Phylum** | **Genus** | **Species** |
| --- | --- | --- | --- | --- | --- |
| hypothetical protein | 0.29 | 0.01 | Bacteroidetes | Bacteroides | Bacteroides sp. UNK.MGS-14 |
| TCP-1/cpn60 chaperonin family protein, partial | 0.30 | 0.01 | Proteobacteria | Enterobacter | Enterobacter hormaechei |
| 30S ribosomal protein S9 | 0.30 | 0.01 | Proteobacteria | Salmonella | Salmonella enterica |
| ribosomal protein L16 | 0.32 | 0.01 | Proteobacteria | Enterobacter | Enterobacter hormaechei |
| PL29 family lyase N-terminal domain-containing protein, partial | 0.35 | 0.00 | Bacteroidetes | Bacteroides | Bacteroides dorei |
| PL29 family lyase N-terminal domain-containing protein | 0.27 | 0.02 | Bacteroidetes | Bacteroides | Bacteroides vulgatus |
| phosphopyruvate hydratase | 0.39 | 0.00 | Firmicutes | Holdemania | Holdemania sp. Marseille-P2844 |
| MULTISPECIES: 50S ribosomal protein L7/L12 | 0.24 | 0.05 | Firmicutes | Blautia | [Ruminococcus] torques |
| porin OmpA | 0.26 | 0.03 | Proteobacteria | Escherichia | Escherichia coli |
| phosphoglycerate kinase, partial | 0.39 | 0.00 | Proteobacteria | Enterobacter | Enterobacter cloacae |
| 50S ribosomal protein L15, partial | 0.38 | 0.00 | Proteobacteria | Escherichia | Escherichia coli |
| MULTISPECIES: elongation factor G | 0.25 | 0.04 | Firmicutes | Ruminococcus | Ruminococcus sp. UNK.MGS-30 |
| MULTISPECIES: 50S ribosomal protein L4 | 0.35 | 0.00 | Proteobacteria | Morganella | Morganella sp. GLFB |
| porin, partial | 0.30 | 0.01 | Proteobacteria | Escherichia | Escherichia coli |
| formate acetyltransferase, partial | 0.26 | 0.03 | Proteobacteria | Klebsiella | Klebsiella pneumoniae |
| MULTISPECIES: alkyl hydroperoxide reductase subunit C | 0.39 | 0.00 | Proteobacteria | Enterobacter | Enterobacter asburiae |
| MULTISPECIES: type I glyceraldehyde-3-phosphate dehydrogenase | 0.27 | 0.02 | Firmicutes | Blautia | [Ruminococcus] torques |
| chaperonin GroEL | 0.27 | 0.02 | Proteobacteria | Citrobacter | Citrobacter amalonaticus |
| pyruvate:ferredoxin (flavodoxin) oxidoreductase | 0.26 | 0.03 | Firmicutes | Coprococcus | Coprococcus catus |
| phosphopyruvate hydratase, partial | 0.23 | 0.05 | Proteobacteria | Enterobacter | Enterobacter hormaechei |
| aldehyde dehydrogenase family protein, partial | 0.32 | 0.01 | Proteobacteria | Citrobacter | Citrobacter amalonaticus |
| MULTISPECIES: elongation factor Tu | 0.30 | 0.01 |  |  |  |
| elongation factor Tu, partial | 0.34 | 0.00 | Proteobacteria | Morganella | Morganella morganii |
| MULTISPECIES: autonomous glycyl radical cofactor GrcA | 0.28 | 0.02 | Proteobacteria | Citrobacter | Citrobacter amalonaticus |
| RagB/SusD family nutrient uptake outer membrane protein, partial | 0.31 | 0.01 | Bacteroidetes | Porphyromonas | Porphyromonas sp. 31_2 |
| MULTISPECIES: histone-like nucleoid-structuring protein H-NS | 0.33 | 0.00 | Proteobacteria | Enterobacter | Enterobacter asburiae |
| phosphopyruvate hydratase | 0.23 | 0.05 | Firmicutes | Eubacterium | Eubacterium sp. CAG:274 |
| MAG: flagellar hook protein | 0.24 | 0.04 | Firmicutes | Eubacterium | Eubacterium sp. 41_20 |
| hypothetical protein | 0.28 | 0.02 | Bacteroidetes | Bacteroides | Bacteroides vulgatus |
| molecular chaperone DnaK, partial | 0.31 | 0.01 | Proteobacteria | Klebsiella | Klebsiella oxytoca |
| phosphoenolpyruvate carboxykinase [ATP] | 0.25 | 0.04 | Firmicutes | Roseburia | Roseburia sp. CAG:45 |
| 50S ribosomal protein L1, partial | 0.35 | 0.00 | Proteobacteria | Citrobacter | Citrobacter amalonaticus |
| ethanolamine utilization microcompartment protein EutM | 0.25 | 0.03 |  |  |  |
| DUF6383 domain-containing protein | 0.30 | 0.01 | Bacteroidetes | Parabacteroides | Parabacteroides distasonis |
| MULTISPECIES: phosphoenolpyruvate-protein phosphotransferase PtsI | 0.28 | 0.02 | Proteobacteria | Enterobacter | Enterobacter cloacae |
| aspartate ammonia-lyase | 0.27 | 0.02 | Proteobacteria | Enterobacter | Enterobacter hormaechei |
| ABC transporter substrate-binding protein | 0.25 | 0.04 | Firmicutes | Blautia | [Ruminococcus] gnavus |
| autonomous glycyl radical cofactor GrcA, partial | 0.24 | 0.04 | Proteobacteria | Klebsiella | [Enterobacter] aerogenes |
| substrate-binding domain-containing protein | 0.24 | 0.04 | Firmicutes | Blautia | [Ruminococcus] gnavus |
| MULTISPECIES: triose-phosphate isomerase | 0.31 | 0.01 | Proteobacteria | Citrobacter | Citrobacter amalonaticus |
| S-layer homology domain-containing protein | 0.31 | 0.01 | Firmicutes | Oscillibacter | Oscillibacter sp. KLE 1728 |
| elongation factor G | 0.36 | 0.00 | Proteobacteria | Enterobacter | Enterobacter kobei |
| iron-containing alcohol dehydrogenase | 0.32 | 0.01 | Proteobacteria | Citrobacter | Citrobacter amalonaticus |
| MULTISPECIES: DUF6383 domain-containing protein | 0.33 | 0.00 | Bacteroidetes | Parabacteroides | Parabacteroides distasonis |
| hypothetical protein | 0.26 | 0.03 | Firmicutes | Faecalibacterium | Faecalibacterium prausnitzii |
| porin OmpA | 0.24 | 0.05 | Proteobacteria | Citrobacter | Citrobacter freundii |
| MULTISPECIES: 50S ribosomal protein L22 | 0.37 | 0.00 | Proteobacteria | Enterobacter | Enterobacter hormaechei |
| PTS system mannose/fructose/sorbose family transporter subunit IID | 0.30 | 0.01 | Firmicutes | Faecalicoccus | Faecalicoccus pleomorphus |

**Supplemental Table 5.** Negative correlation of bacterial proteins with 90-day mortality (data for Figure 4D). Correlation coefficient (R) was calculated using spearman correlation. Proteins were selected for further analysis if R<-0.2.

| **Protein name** | ***R*** | ***P-*value** | **Phylum** | **Genus** | **Species** |
| --- | --- | --- | --- | --- | --- |
| elongation factor G | -0.28 | 0.02 | Firmicutes | Eubacterium | [Eubacterium] eligens |
| hypothetical protein, partial | -0.24 | 0.04 | Bacteroidetes | Bacteroides | Bacteroides uniformis |
| MULTISPECIES: 30S ribosomal protein S10 | -0.28 | 0.02 | Firmicutes | Streptococcus | Streptococcus agalactiae |
| MULTISPECIES: oleate hydratase | -0.25 | 0.03 | Firmicutes |  | Clostridiales bacterium VE202-27 |
| NADH peroxidase | -0.26 | 0.03 | Firmicutes | Romboutsia | Romboutsia timonensis |
| MULTISPECIES: hypothetical protein | -0.26 | 0.03 | Firmicutes | Blautia | Blautia sp. CAG:257 |
| tagaturonate reductase | -0.25 | 0.04 | Firmicutes | Eubacterium | Eubacterium sp. CAG:38 |
| twin-arginine translocation signal domain-containing protein | -0.27 | 0.02 | Firmicutes | Faecalibacterium | Faecalibacterium prausnitzii |
| acyl-CoA dehydrogenase family protein | -0.24 | 0.04 | Firmicutes | Clostridium | uncultured Clostridium sp. |
| GGGtGRT protein | -0.24 | 0.04 | Firmicutes | Blautia | [Ruminococcus] torques |
| phosphopyruvate hydratase | -0.27 | 0.02 | Firmicutes | Streptococcus | Streptococcus gallolyticus |
| fructose-bisphosphate aldolase | -0.25 | 0.03 | Firmicutes | Streptococcus | Streptococcus salivarius |
| 30S ribosomal protein S7 | -0.30 | 0.01 | Bacteroidetes | Prevotella | Prevotella fusca |
| chaperonin GroEL, partial | -0.27 | 0.02 | Firmicutes | Dorea | Dorea longicatena |
| hypothetical protein | -0.27 | 0.02 | Firmicutes | Eubacterium | [Eubacterium] eligens |
| elongation factor Tu | -0.32 | 0.01 | Firmicutes | Clostridium | uncultured Clostridium sp. |
| PEP/pyruvate-binding domain-containing protein | -0.36 | 0.00 | Firmicutes | Ruminococcus | Ruminococcus callidus |
| glutamine synthetase III | -0.37 | 0.00 | Firmicutes | Roseburia | Roseburia faecis |
| short-chain-enoyl-CoA hydratase | -0.29 | 0.01 | Firmicutes | Oscillibacter | Oscillibacter sp. CAG:241_62_21 |
| acyl-CoA dehydrogenase [ | -0.24 | 0.04 | Firmicutes |  | Clostridia bacterium UC5.1-1A9 |
| phosphopyruvate hydratase | -0.24 | 0.04 | Firmicutes | Merdibacter | Merdibacter massiliensis |
| - | -0.27 | 0.02 | Firmicutes | Lachnospira | Lachnospira pectinoschiza |
| gluconate 5-dehydrogenase | -0.37 | 0.00 | Firmicutes | Eubacterium | [Eubacterium] eligens |
| 3-hydroxyacyl-CoA dehydrogenase NAD-binding domain-containing protein | -0.37 | 0.00 | Firmicutes | Coprococcus | Coprococcus eutactus |
| MULTISPECIES: L-fucose isomerase | -0.29 | 0.01 | Firmicutes | Blautia | Blautia sp. CAG:257 |
| flagellin | -0.25 | 0.04 | Firmicutes | Lachnospira | Lachnospira pectinoschiza |
| 50S ribosomal protein L10 | -0.31 | 0.01 | Firmicutes |  | Lachnospiraceae bacterium AC2014 |
| succinate CoA transferase | -0.24 | 0.05 |  |  |  |
| twin-arginine translocation signal domain-containing protein | -0.24 | 0.04 | Firmicutes | Faecalibacterium | uncultured Faecalibacterium sp. |
| MULTISPECIES: 50S ribosomal protein L7/L12 | -0.23 | 0.05 | Firmicutes | Clostridium | Clostridium sp. CAG:127 |
| MULTISPECIES: 50S ribosomal protein L34 | -0.31 | 0.01 |  |  |  |
| sn-glycerol-3-phosphate ABC transporter ATP-binding protein UgpC | -0.24 | 0.04 |  |  |  |
| ketose-bisphosphate aldolase | -0.25 | 0.04 |  |  |  |
| triose-phosphate isomerase | -0.31 | 0.01 | Firmicutes | Fusicatenibacter | Fusicatenibacter saccharivorans |
| MULTISPECIES: 50S ribosomal protein L16 | -0.24 | 0.04 |  |  |  |
| sugar kinase | -0.24 | 0.04 | Firmicutes | Eubacterium | [Eubacterium] eligens |
| class II fructose-1,6-bisphosphate aldolase | -0.24 | 0.04 | Firmicutes | Roseburia | Roseburia sp. CAG:10041_57 |
| acyl-CoA dehydrogenase | -0.25 | 0.04 | Firmicutes | Butyrivibrio | Butyrivibrio crossotus |
| NADH peroxidase | -0.23 | 0.05 |  |  |  |
| bifunctional 4-hydroxy-2-oxoglutarate aldolase/2-dehydro-3-deoxy-phosphogluconate aldolase | -0.34 | 0.00 | Firmicutes | Lachnospira | Lachnospira pectinoschiza |
| flagellin | -0.25 | 0.03 | Firmicutes | Roseburia | Roseburia inulinivorans |
| RagB/SusD family nutrient uptake outer membrane protein, partial | -0.25 | 0.04 | Bacteroidetes | Bacteroides | Bacteroides oleiciplenus |
| 50S ribosomal protein L5 | -0.29 | 0.01 | Firmicutes | Eubacterium | Eubacterium sp. CAG:38 |
| ketol-acid reductoisomerase | -0.23 | 0.05 | Firmicutes | Eubacterium | Eubacterium sp. CAG:274 |
| sn-glycerol-3-phosphate ABC transporter ATP-binding protein UgpC | -0.26 | 0.03 |  |  |  |
| pyruvate-flavodoxin oxidoreductase | -0.27 | 0.02 | Firmicutes | Lachnospira | Lachnospira pectinoschiza |
| phosphoglycerate kinase | -0.25 | 0.03 | Firmicutes | Clostridium | uncultured Clostridium sp. |
| NADP-specific glutamate dehydrogenase | -0.30 | 0.01 | Firmicutes | Lachnospira | Lachnospira pectinoschiza |
| 30S ribosomal protein S8 | -0.30 | 0.01 | Firmicutes | Roseburia | Roseburia intestinalis |
| phosphoglycerate kinase | -0.26 | 0.03 |  |  |  |
| discoidin domain-containing protein | -0.26 | 0.03 | Firmicutes | Ruminococcus | uncultured Ruminococcus sp. |
